# Supplementary material for: CSF1R ligands promote microglial proliferation but are not the sole regulators of developmental microglial proliferation
Source: Development. 2025 Jun 3;152(20):dev204610. doi: 10.1242/dev.204610 (PMC12188242; doi:10.1242/dev.204610)
Supplement: Supplementary information [file develop-152-204610-s1.pdf]

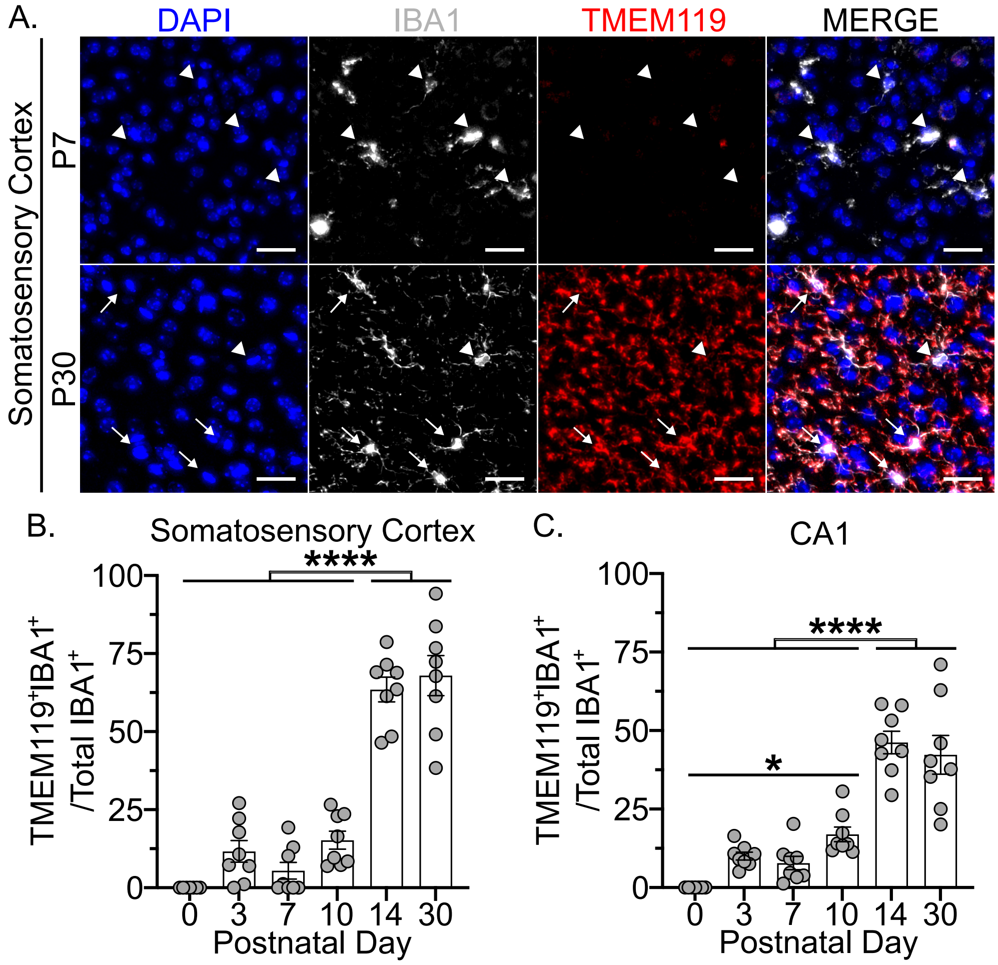

Fig. S1. TMEM119 expression is negligible in the developing somatosensory cortex and CA1 prior to P14.

A. Representative images for IBA1 (grey) and TMEM119 (red) expression at P7 and P30 in the somatosensory cortex. White arrow heads = $IBA1^{+}TMEM119^{-}$, white arrows = $IBA1^{+}TMEM119^{+}$. Scale bar = 50 μm.
B. Plot of the mean percentage of the total $IBA1^{+}$ cells that are also $TMEM119^{+}$ in the somatosensory cortex at each developmental timepoint.
C. Plot of the mean percentage of the total $IBA1^{+}$ cells that are also $TMEM119^{+}$ in the CA1 at each developmental timepoint.
D. Bars represent mean±s.e.m. ; B-C: n = 7-8 mice per timepoint.

Development • Supplementary information

A.

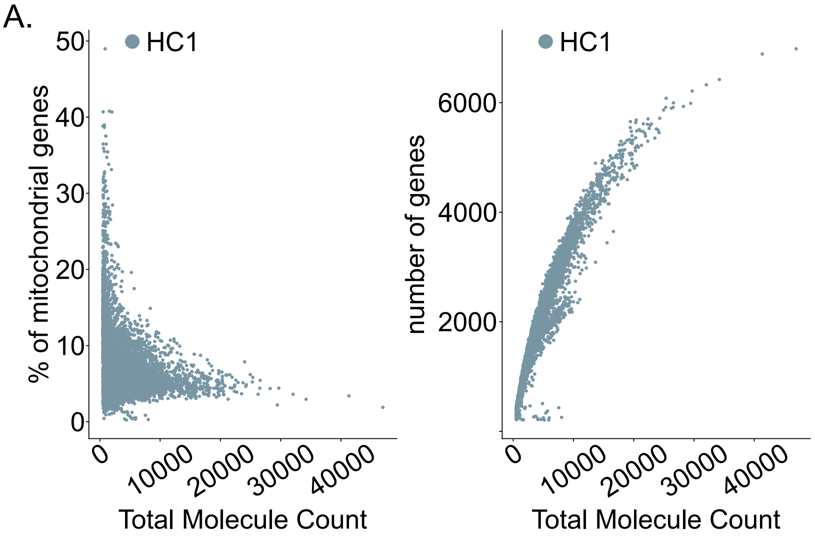

B.

	Mitochondrial Gene %	Total Gene Count
HC1	<10	5000
HC2	<10	3000
CX1	<10	5000
CX2	<10	3000

C.

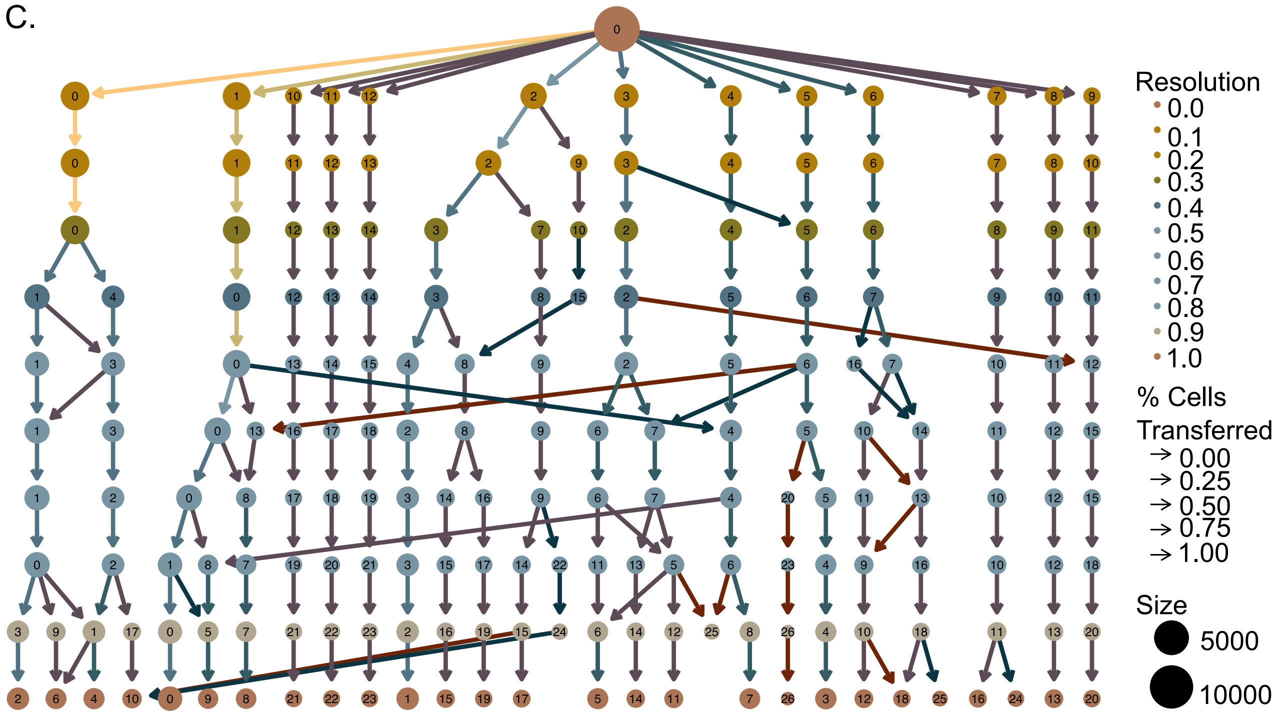

Development • Supplementary information

Fig. S2. Quality control and clustering refinement for P7 cortex and hippocampus datasets.

A. Plot of the percentage of mitochondrial genes relative to the total molecule count in the P7 hippocampus to identify dead and/or dying cells (left). Plot of the number of genes relative to the total molecule count in the P7 hippocampus to identify and exclude doublets and multiplets (right).
B. Table of mitochondrial gene percentage cutoffs (<10%) to identify live, and total gene number cutoffs (<3000 or <5000) to identify single, cells for each sample.
C. Clustering tree to identify the optimal clustering resolution between 0.1 and 1.0. The size of the circle indicates cluster size and arrow opacity indicates the probability of cell transfer between clusters.

Development • Supplementary information

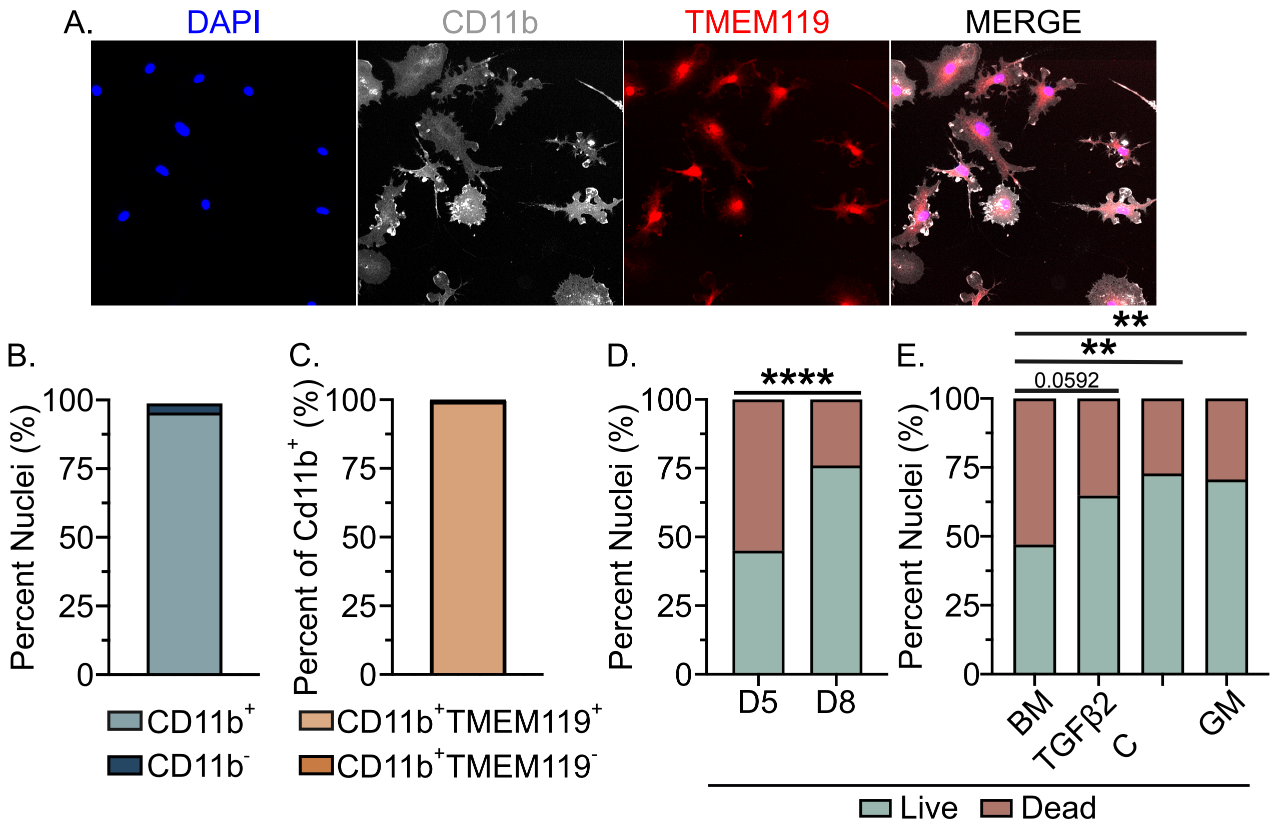

Fig. S3. Immunopanned, serum-free primary microglia are highly pure and viable.

A. Representative images for CD11b (grey) and TMEM119 (red) expression in cultured microglia at day 7 *in vitro*. Scale bar = 50 µm.
B. Stacked bar graph for the proportion of all nuclei that are $CD11b^{+}$ in immunopanned microglia at D8 *in vitro*.
C. Stacked bar plot of microglial purity depicting the percentage of nuclei that are $CD11b^{+}$ and $CD11b^{-}$.
D. Stacked bar plot for the proportion of all $CD11b^{+}$ cells that are either $TMEM119^{+}$ or $TMEM119^{-}$.
E. Stacked bar plot of microglial viability depicted by calceinAM (live cells) and propidium iodide labeled nuclei (dead cells) at day 5 and day 8 in culture.
F. Stacked bar plot of microglial viability depicted by calceinAM (live cells) and propidium iodide labeled nuclei (dead cells) with different compositions of microglial growth media components. Treatments were added at day five *in vitro* for an additional three days before assessments.

Development • Supplementary information

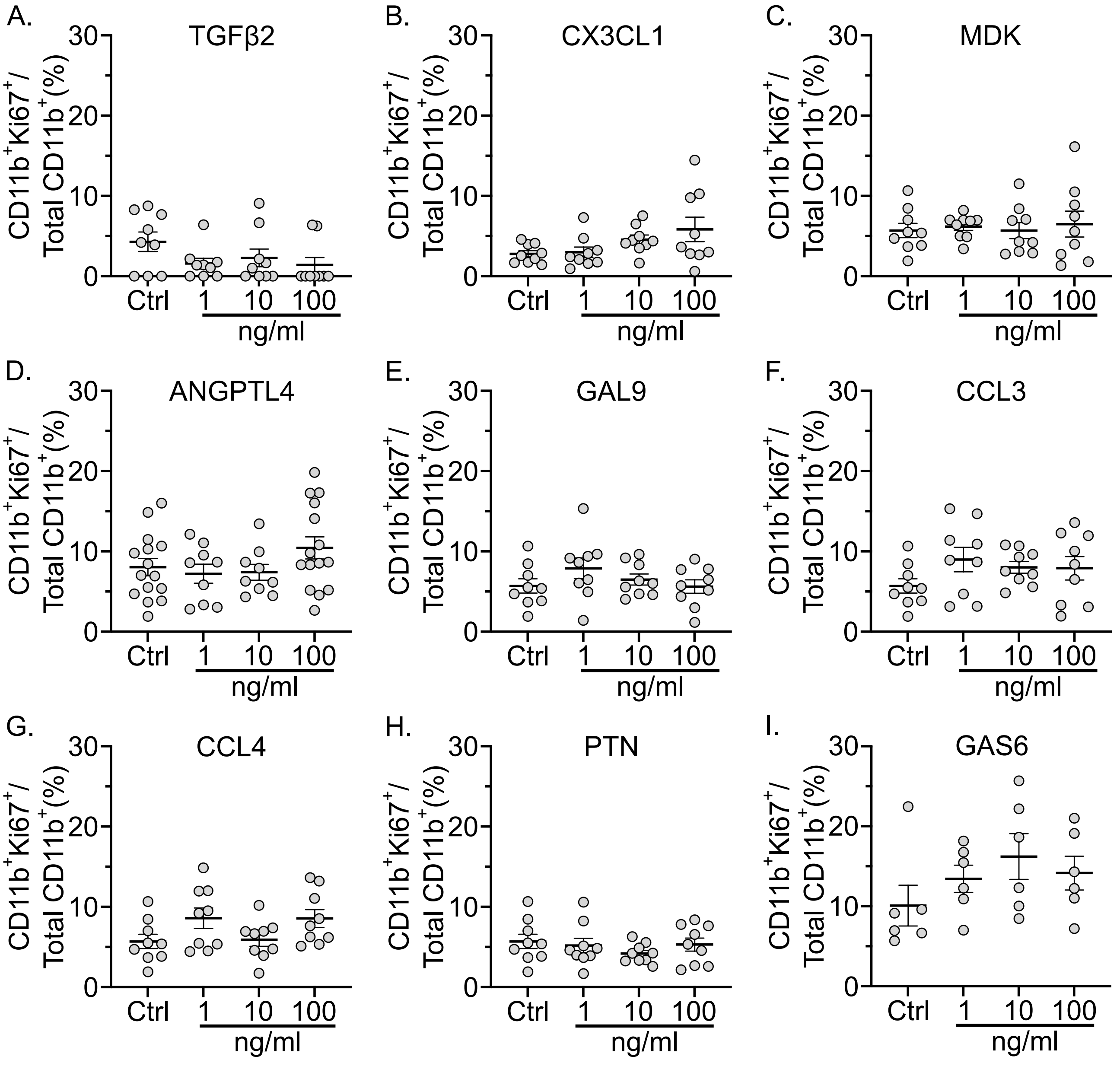

Development • Supplementary information

Fig. S4. Immunopanned microglia do not proliferate in response to most ligands predicted to signal onto microglia at P7.

A. Dot plot of the percentage of cultured microglia that are proliferative with increasing concentrations of TGF-β2.
B. Dot plot of the percentage of cultured microglia that are proliferative with increasing concentrations of CX3CL1.
C. Dot plot of the percentage of cultured microglia that are proliferative with increasing concentrations of MDK.
D. Dot plot of the percentage of cultured microglia that are proliferative with increasing concentrations of ANGPTL4.
E. Dot plot of the percentage of cultured microglia that are proliferative with increasing concentrations of GAL9.
F. Dot plot of the percentage of cultured microglia that are proliferative with increasing concentrations of CCL3.
G. Dot plot of the percentage of cultured microglia that are proliferative with increasing concentrations of CCL4.
H. Dot plot of the percentage of cultured microglia that are proliferative with increasing concentrations of PTN.
I. Dot plot of the percentage of cultured microglia that are proliferative with increasing concentrations of GAS6.

Bars represent mean±s.e.m., A-L n=2-3 independent microglial cultures with treatments in triplicate. D-L: microglia were cultured for five days in microglial growth media before treatments were added for an additional three days prior to assessment.

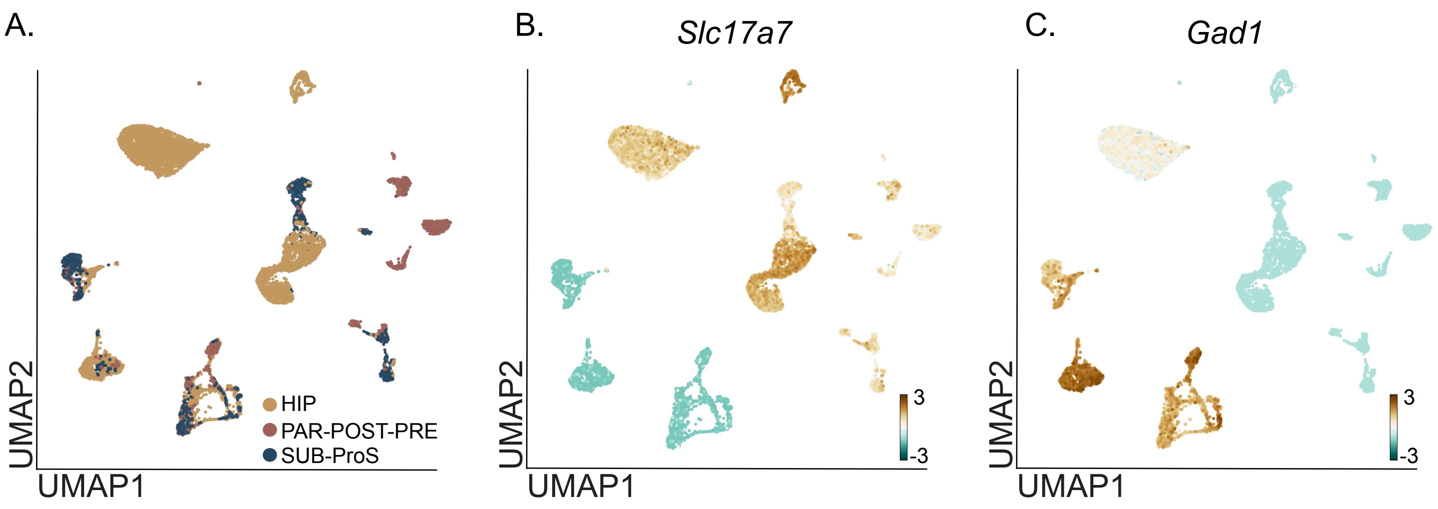

Fig. S5. Excitatory and inhibitory neurons are present throughout the hippocampus

A. UMAP plot depicting the regional distribution of cell populations hippocampus (HIP), parasubiculum, postsubiculum, presubiculum (PAR-POST-PRE), and subiculum and prosubiculum (SUB-ProS). Dots represents individual cells clustered based on transcriptional similarity, while colours indicate the regions (HIP, PAR-POST-PRE, and SUB-ProS).
B. UMAP plot depicting expression of *Slc17a7* in the adult hippocampus to identify excitatory neurons.
C. UMAP plot depicting expression of *Gad1* in the adult hippocampus to identify inhibitory neurons.
